# The complete replicons of 16 *Ensifer meliloti* strains offer insights into intra- and inter-replicon gene transfer, transposon-associated loci, and repeat elements

**DOI:** 10.1099/mgen.0.000174

**Published:** 2018-04-19

**Authors:** Matthew Nelson, Joseph Guhlin, Brendan Epstein, Peter Tiffin, Michael J. Sadowsky

**Affiliations:** ^1^​ Biotechnology Institute and Department of Soil, Water, and Climate, University of Minnesota, St. Paul, MN 55108, USA; ^2^​ Department of Plant and Microbial Biology, University of Minnesota, St. Paul, MN 55108, USA

**Keywords:** genome evolution, structural variation, repeat and IS elements, accessory plasmids, comparative genomics, rhizobia

## Abstract

*Ensifer meliloti* (formerly *Rhizobium meliloti* and *Sinorhizobium meliloti*) is a model bacterium for understanding legume–rhizobial symbioses. The tripartite genome of *E. meliloti* consists of a chromosome, pSymA and pSymB, and in some instances strain-specific accessory plasmids. The majority of previous sequencing studies have relied on the use of assemblies generated from short read sequencing, which leads to gaps and assembly errors. Here we used PacBio-based, long-read assemblies and were able to assemble, *de novo*, complete circular replicons. In this study, we sequenced, *de novo-*assembled and analysed 10 *E. meliloti* strains. Sequence comparisons were also done with data from six previously published genomes. We identified genome differences between the replicons, including mol% G+C and gene content, nucleotide repeats, and transposon-associated loci. Additionally, genomic rearrangements both within and between replicons were identified, providing insight into evolutionary processes at the structural level. There were few cases of inter-replicon gene transfer of core genes between the main replicons. Accessory plasmids were more similar to pSymA than to either pSymB or the chromosome, with respect to gene content, transposon content and G+C content. In our population, the accessory plasmids appeared to share an open genome with pSymA, which contains many nodulation- and nitrogen fixation-related genes. This may explain previous observations that horizontal gene transfer has a greater effect on the content of pSymA than pSymB, or the chromosome, and why some rhizobia show unstable nodulation phenotypes on legume hosts.

## Data Summary

All genomes were deposited in GenBank under Bioproject PRJNA388336 with the following accession numbers: HM006_ SRR6032557, SRR6032556, SRR6032555, SRR6032554; KH35C_ SRR6032553; KH46_ SRR6032552; M162_ SRR6032551; M270_ SRR6032550; Rm41_ SRR6032549; T073_ SRR6032548; USDA1021_ SRR6032559; USDA1106_ SRR6032558; USDA1157_ SRR6032560.

**Impact Statement**

This article provides evidence that the three main replicons of *Ensifer meliloti* have distinct gene content and gene patterns that are maintained even as strains genetically diverge. The symbiotically important replicon, pSymA, appears to preferentially recombine or transfer genes with the accessory plasmids. Some accessory plasmids were previously shown to be involved in host specificity. This suggests a mechanism for gaining or losing genes involved in symbiosis and host selection. The replication protein for the accessory plasmids, RepA, is found in numerous other bacterial species and may have been acquired by plasmid transfer from other soil microbiota. Our results reveal that some structural rearrangements of replicons in *E. meliloti* are common, but that gene translocation between replicons is relatively rare, or selected against. Intra-replicon gene transfer is associated with repeat elements, but not transposon-associated loci. The gene transfer events that occurred between the accessory plasmids and pSymA in *E. meliloti* demonstrate one mechanism by which the *Ensifer–*legume symbiosis is constantly evolving. Overall, and despite what has been found in other rhizobia, *E. meliloti* has a fairly stable genome structure on an evolutionary timescale.

## Introduction

Rhizobia are an important group of bacteria because of the symbioses they form with legume plants. These bacteria provide the plant with fixed nitrogen by converting atmospheric N_2_ into a plant-usable form. In exchange, the plant provides carbon to the rhizobia located within root or stem nodules, thereby supporting greater bacterial growth and reproduction. The *Medicago truncatula–Ensifer meliloti* (*Sinorhizobium meliloti*) symbiosis is a model system to better understand the genetic basis and evolution of rhizobial–legume symbioses and the N_2_-fixation process [1]. *E. meliloti* strains contain more than one large replicon, which is similar to roughly 10 % of assayed bacterial species [2]. In *E. meliloti* large non-chromosomal replicons are referred to as megaplasmids or symbiotic (Sym) plasmids [2].

The *E. meliloti* reference genome, Rm1021, has a chromosome and two megaplasmids, pSymA and pSymB, but not other small accessory plasmids [1]. Previous work has shown that genes involved in similar functions tend to be concentrated on particular replicons: pSymA contains genes playing essential roles in symbiosis, including nodule formation and symbiotic nitrogen fixation [1], whereas pSymB contains a large proportion of genes involved in import/export functions [3], and the chromosome contains most of the housekeeping genes [4–6]. These replicon-specific gene functions have been hypothesized to be the result of the initial acquisition of plasmids followed by horizontal gene transfer events [6]. Some strains also contain smaller accessory plasmids [7], some of which have been shown to affect nodulation and metabolic potential [8, 9].

Previous studies have shown that the three main replicons of *E. meliloti* have distinct evolutionary histories [10], and inter-replicon differences can be seen in the: (1) levels of standing nucleotide variation [10], (2) effects of purifying and positive selection [5, 11], (3) proportion of duplicated and horizontally transferred genes [11], and (4) structural rearrangements and core gene content [5]. These studies have relied primarily on the use of short-read sequencing (mainly Illumina-based Hiseq or Miseq platforms) and a limited number of complete genomes. However, it is well known that mapping short-read sequences back to a single reference genome biases assembly and downstream analyses to what was found in the reference genome, and thus provides limited insight into large structural variation, genes missing from the reference genome, intra-replicon gene movement and quantification of repeated sequences [12, 13].

Fully assembled, reference-quality genomes generated from long-read technologies, such as Pacific Biosciences (PacBio) sequencing [14], allow for better assignment of genome rearrangements, repetitive sequences, gene content and evolutionary histories of populations. This enables characterization of transposon-associated loci (TALs), genes that encode proteins that may mediate the transposition and duplications of DNA within the genome [15], and repeat elements (REs), sequences of DNA repeated one or more times within a single genome. These features are difficult to assemble and differentiate using short-read data. The TALs and REs have been inferred to facilitate roles in gene movement among bacterial lineages [15], and presumably facilitate movement of genic regions between replicons [16].

Accessory plasmids are small replicons present in some, but not all, *E. meliloti* strains [7, 9, 13] and some have been identified as facilitating important biological roles such as metabolic potential, host incompatibility and nodulation competitiveness [9]. Relatively little is known about the origin or evolution of accessory plasmids in *Ensifer*, although they are generally thought to be transient components (easily gained or lost) from the species pan-genome. The limited data on *E. meliloti* accessory plasmids suggest that genes found on these plasmids are similar to those found on pSymA or pSymB. However, no extensive genomic analyses of multiple plasmids have been done [but see 7, 13, 17], and reference-quality genomes can provide data for such analyses.

Here we describe the complete genome sequence of 16 *E. meliloti* reference-quality genomes, 10 of which were newly sequenced in this study using high-coverage PacBio data. The fully assembled genome sequences were used to characterize: (1) the diversity of gene content, TALs and REs found in *E. meliloti*, (2) gene transfer events between replicons and (3) genomic composition and relationships among *E. meliloti* accessory plasmids. Our research shows that there is benefit in analysing *E. meliloti* in a replicon-independent manner, and describes how *E. meliloti* can gain and lose genes in a manner consistent with maintenance of overall genome stability and functionality.

## Methods

### Genome sequencing, assembly and annotation

Genomic DNA from the 10 *E. meliloti* strains was isolated by using UltraClean Microbial DNA Isolation Kits from MoBio Laboratories. Strains were previously obtained [18] or newly acquired from the USDA culture collection. Cultures were grown at 30 °C in TY medium [19]. Genomic sequence data were generated using a Pacific Biosciences (PacBio) RS II sequencer at the Mayo Clinic with one PacBio single molecule real time (SMRT) cell per strain. Genomes were assembled using hgap version 3.0 [20]. Each genome was assembled multiple times, adjusting the predicted size of the genome during each subsequent assembly run. The genomes were circularized and replicons were confirmed using gepard version 1.3.1 [21]: coverage range, 33.3–153.6; read range, 20 333–100 711; N50 range, 17 362–20 321; and average read length range, 10 826–20 341. The assembled genomes were individually polished with Pilon 1.16 [22], using Illumina reads from previous studies that were mapped to the PacBio assemblies using bwa version 0.7.12-r1039, and using the ‘mem’ algorithm [23]. Files were converted from SAM to BAM files using samtools view 1.3 [24]. Pilon was run with the required arguments and additionally ‘—changes —fix "bases" '. The complete commands for running bwa, samtools and Pilon on the M2 genome are available at: https://github.com/jguhlin/pacbio-paper-code/blob/master/pilon-M2-genome/run-pilon.sh. Base pair changes during the polishing stage for each assembly ranged from 4 to 624 (<0.01 % of the genome) [22]. The protein-coding genes of each assembled genome were predicted using prodigal, with no specialized parameters [25]. Replicon names were assigned based on sequence similarity to the reference strain *E. meliloti* Rm2011 [26]. The six previously sequenced strains were imported from NCBI, and all genomes were also independently assembled and annotated using the MaGE and the MicroScope platform (http://www.genoscope.cns.fr/agc/microscope/home/index.php). Annotations are also available at: https://github.com/jguhlin/pacbio-paper-code/tree/master/gene-prediction.

### Identification of syntenic regions, core and pan genomes

Synteny analyses was performed using nucmer from the mummer package (version 3.1) [27]. Plots and downstream analyses were done using custom code. Core and pan genomes were generated by performing an all-vs-all blast+ comparison on the predicted protein sequences, clustering based on blast+ bitscores using mcl with an inflation value of 10.0, due to strains belonging to a single species. Orthology-based approaches are not appropriate for this analysis [28, 29]. Identification of single-copy core genes was performed using an ODG database and a cypher query, available at the Github repository referenced below, with additional analysis with custom code [30]. Custom code, commands and scripts are available at: http://github.com/jguhlin/pacbio-paper-code. The ‘Pan/Core-Genome’ and the ‘Gene Phyloprofile’ tool of the MicroScope platform was also used to identify similar genes between bacterial strains and individual replicons, with thresholds set at the recommended MicroScope specifications of 80 % amino acid identity and 80 % alignment coverage [31, 32]. The MicroScope protein-coding gene annotations were used to generate core-genome and core-replicon gene content. Inter-replicon gene movements were defined at genes present in the core-genome, but absent in the core-replicon for the chromosome, pSymA and pSymB.

### Identification of transposon-associated elements and repetitive elements

To predict TALs, *de novo* gene prediction was performed on each genome using Prodigal v2.6.3, and exporting coding sequences, peptide sequences and a GFFv3 file detailing predicted genes. The specific commands used are available at:https://gist.github.com/jguhlin/67811311c36e35b0c1ac2ef772c129cb [25]. Functional predictions were generated using orthologous functional prediction via eggNOG v4.5.1 and matched using hmmer v3.1b2 [33, 34]. The TALs were identified based on matching one or more eggNOG-based annotations associated with transposable elements. TALs were subsequently clustered using mcl v14–137 based on sequence similarity bit scores determined by blast+ [28, 29, 35].

Repeats and repeated elements were identified using nucmer, from the mummer version 3.23 package [27], as follows. Total genomic content of each strain was compared against itself (using the command ‘*nucmer –p ID-vs-ID –maxmatch –nosimplify*’ *ID.fasta ID.fasta,* where ID is the strain ID), and the sequences of matches were extracted and combined with the matches from all strains. nucmer was subsequently used to compare all repeats to each other, and mcl was used to cluster repeats based on sequence matching coverage pairwise for each repeat [35]. Repeats were aligned using mafft (with arguments —*maxiterate 1000 —localpair –adjustdirection*), and poor alignments were trimmed or removed using TrimAL (*-resoverlap 0.6 -seqoverlap 60*). Repeats were re-aligned with mafft, using the same arguments as previously [36, 37]. hmm profiles were built from these alignments using hmmer [34], and the genome of each strain was analysed for repeat content using nhmmscan, with an e-value cutoff of 0.0001 [34]. Regions matching multiple hmm profiles were assigned to the best match, by either length, identity percentage or score, in that order. Principal component analyses (PCAs) were performed on both the repetitive element and the transposable-associated element contents of each replicon in each strain [38].

### Mantel test

Correlations among pairwise genetic distance matrices were tested for each replicon. A genetic distance matrix for each replicon was constructed using the concatenated alignments of single copy core genes and the dist.dna function from the r [39] package ape [39]. This was done using the TN93 model of evolution [40]. A Mantel test [41] was implemented in the ade4 package [39], with 10 000 permutations, to calculate the correlation between distance matrices from different replicons and to test for significance.

### Relationships among RepA sequences

The relationships among the RepA protein-coding genes from several bacterial species were characterized by constructing a maximum-likelihood phylogeny using the PhyML implementation on thewww.phylogeny.fr webserver, a part of Méthodes et algorithmes pour la bio informatique [42–48].

## Results

### Replicon overview

All replicons in the 10 new strains sequenced in this study were *de novo* and completely assembled, and fully circularized using both PacBio and Illumina sequencing data. Our analyses were supplemented with six previously published complete genomes [1, 13, 26, 49, 50]. The total sizes of genomes from these 16 strains ranged from 6.68 to 7.27 Mb (Tables 1 and S1, available in the online version of this article). The strains were obtained from geographically diverse regions of the USA, Europe, Australia and the Middle East (Table S1). Each genome contained three main replicons – a chromosome and the two megaplasmids, pSymA and pSymB – and in many cases from one to three accessory plasmids. The chromosomes had an average G+C content of 62.72 mol% – similar to pSymB, which had an average of 62.40 mol% (pairwise *t*-test for difference in G+C content, *P*=0.34 after Bonferroni correction). In contrast, replicon pSymA had substantially lower G+C content, 60.31 mol% (*t*-test *P*<0.001 compared to the chromosome and pSymB), similar to the 59.18 mol% average of the accessory plasmids. The accessory plasmids showed great variance in G+C content, with σ^2^_accessory_ = 0.888, σ^2^_psyma_ = 0.020, σ^2^_psymb_ = 0.119 and σ^2^_main_ = 0.0052, perhaps reflecting their diverse origins.

**Table 1.** Genomic properties of the chromosomes, megaplasmids and accessory plasmids from 16 characterized *E. melioti* strains For each entry, the range of values is presented in parentheses below the mean. Detailed data are available in Table S1. While no accessory plasmid exceeded 1 Mb in size, the number of TALs and REs per megabase is presented to allow for a comparison.

Characteristic	Replicon			
	Chromosome	pSymA	pSymB	Accessory
Size (Mb)	3.69 (3.43–3.91)	1.41 (0.89–1.63)	1.69 (1.62–2.01)	0.23 (0.07–0.42)
Number of genes	3807 (3545–4203)	1699 (1439–2100)	1700 (1620–1747)	298 (107–497)
% genes that are core	0.72 (0.69–0.75)	0.45 (0.40–0.46)	0.87 (0.85–0.87)	0
G+C content (mol%)	62.72 (62.57–62.83)	60.31 (59.97–60.54)	62.40 (62.18–62.6)	59.18 (57.45–60.66)
RE number/Mb	431.4 (417.0–446.5)	437.1 (380.0–493.8)	399.2 (390.0–408.3)	571.1 (268.0–853.3)
RE families	464	360	453	289
TAL number/Mb	86.9 (80.4–92.9)	134.9 (109.8–160.3)	115.8 (106.5–141.7)	251.0 (112.5–440.0)
TAL families	281	184	140	205

### General core genome and replicon-specific core genomes

**Fig. 1.**
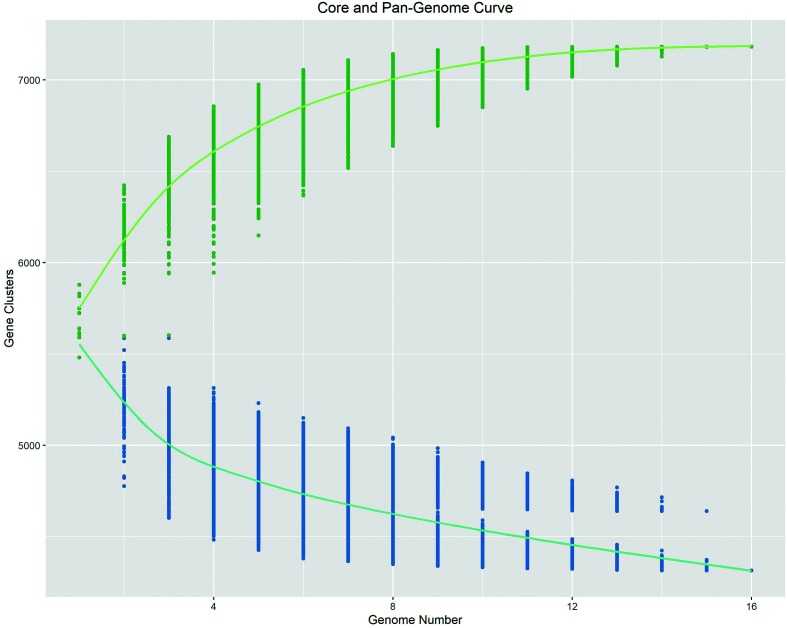
Core- and pan-genome curves for our 16 *E. meliloti* strains. Green represents the pan-genome curve, and each point represents a combination of different genomes. Blue represents the core-genome curve, with a combination of our 16 genomes specified by genome number. Genomes are represented by distinct gene clusters, rather than individual genes.

**Fig. 2.**
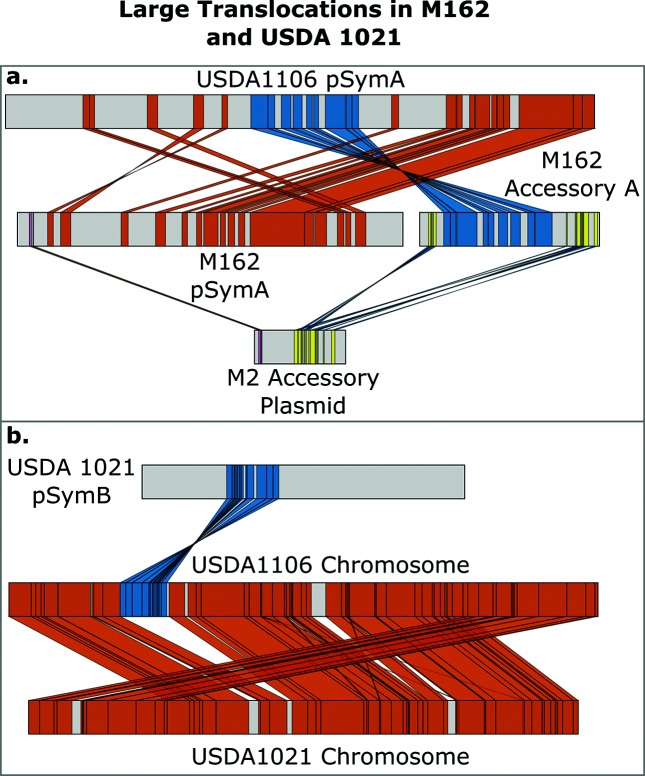
Large translocation events identified in strains M162 and USDA1021, with USDA1106 serving as a typical example of the replicons. (a) *E. meliloti* pSymA appears to have split in strain M162, as identified by a typical pSymA. Furthermore, M162 pSymA shares a replication protein with an accessory plasmid of *E. medicae* strain M2. (b) USDA1021 shows a large translocation from the chromosome to pSymB. For clarity, links shown are as follows: USDA1106–M162 required ≥ 10 kbp and ID ≥ 92 %, while M162–M2 required ≥ 1.5 kbp and ID ≥ 90 %. USDA1106–USDA1021 ≥ 5 kbp and ID ≥ 96.5 %

The core genome of *E. meliloti* was defined by us as consisting of the intersection of all gene families (clusters) found in each of our assayed strains. We clustered genes based on predicted protein similarity from strains: AK83, SM11, BL225c, GR4, RM2011, RM1021, HM006, KH35c, KH46c, M270, RM41, T073, USDA1106, USDA1157, M162 and USDA1021. The core genome of our sample was composed of 4315 gene clusters (Fig. 1). We also performed this analysis using individual replicons, identifying 2472 core gene clusters for the chromosome, 308 for pSymA and 1242 for pSymB (Figs S1–S3). The core genome of the chromosome segments into three distinct patterns due to the inclusion of strains M270 and USDA1021. The pattern resulting from M270 is probably due, for the most part, to a deletion in the chromosome; the chromosome of M270 is the second smallest in our population at 3.5 Mbp. USDA1021 contains a translocation from the chromosome to pSymB, as seen in Fig. 2(b). Fig. S2 segments into two because of a likely split of the pSymA plasmid in strain M162. Because of the large translocations present in strains M162 and USDA1021 (Fig. 2), separate analysis on core genome structure was done excluding these two strains. For this slightly smaller population, 4389 core gene clusters were identified. This accounted for 69–75 % of all genes in the 16 strains (Table S2).

Core genes specific to the chromosome (2734 genes) accounted for 79–87 % of the total genes on each chromosome. Core genes on pSymA (536 genes) accounted for 40–53 % and pSymB (1255 genes) accounted for 85–90 % of the total genes on each replicon (Table S2). The percentage of genes that were part of the core were significantly different between replicons: the chromosome and pSymA (pairwise *t*-tests, Bonferroni-corrected: *P*<2×10^−16^), the chromosome and pSymB (*P*=0.023), and pSymA and pSymB (*P*<2×10^−16^).

### Inter-replicon gene movement

The complete genome assemblies allowed for the identification of inter-replicon gene movement that would not have been identifiable by using short-read sequencing. This is due, in large part, to gaps, potentially unplaced contigs and misplaced contigs due to insertional sequence (IS) elements. To identify inter-replicon gene movement, the gene content for each replicon was compared to those in all strains via blast. Based on the analysis of single-copy core genes, 102 gene translocation events were identified. These were primarily from pSymB to pSymA (62 genes) and the chromosome to pSymB (33 genes). In all cases, the genes were found in 15 strains on an equivalent replicon, and in a single strain on a different replicon. In addition, there was one case where a gene moved from pSymA to pSymB in one strain, but that same gene moved to an accessory plasmid in another strain.

Two large sequence translocation events were identified in *E. meliloti* strains M162 and USDA1021 (Fig. 2). In strain M162, we identified a translocation of 300 kbp from pSymA into a large accessory plasmid. The MaGe annotation for the genes on the accessory plasmid in M162 which match pSymA genes is M162 (pA0019–pA0464). None of these genes was annotated as being *nod*, *nif* or *fix* genes. Moreover, the pSymA in strain M162 had a replication protein (RepA) identical to that found on an accessory plasmid in *Ensifer medicae* strains M2 and WSM419, suggesting a likely horizontal gene transfer event also occurred. RepA is a main replication protein found on non-chromosomal plasmids [51]. Furthermore, the RepA from accessory plasmid M162 was identical to those found on the pSymA replicons in the other *E. meliloti* strains. This indicated that a genomic translocation event involving pSymA and an accessory plasmid occurred in strain M162.

A second large gene transfer event was detected in strain USDA1021, where a 325 kbp region, containing 337 genes, moved from the chromosome to pSymB. The MaGe gene annotations (mpb0525–mpb0862) show these genes are involved in flagella biosynthesis and ATP production. This resulted in a 325 kb increase in the size of pSymB in strain USDA1021 and similar sized reduction in the chromosome as in the other strains (Table S1).

### Transposon-associated loci and repeat elements by replicon

TALs were identified by running a motif search on predicted protein sequences for our strains, and identifying related eggNOG categories. These 1768 eggNOG classifications included transposase, integrase, recombinase, phage integrase family and IS family members (Table S3). About 12 139 TALs were identified in our bacterial population, with 683–981 TALs per genome. The TAL sequences comprised 655–856 kb, accounting for 9.3–11.7 % of the total genome length (Table 1). Of the three main replicons, pSymA had the highest average density of TALs, with a mean 134.9 TALs/Mb. In contrast, pSymB had 115.8 TALs/Mb and the chromosome 86.92 TALs/Mb. The accessory plasmids had an average of 251.0 TALs/Mb, although the accessory plasmids varied widely with a range of 112.5–440 TALs/Mb. Based on sequence identity, the 12 139 TALs were clustered into 474 TAL families, 173 of which were found in every strain, and 42 of which were found in only a single strain. Of the 173 core TALs, 68 were exclusive to a single replicon and 29 were found on all three of the main replicons.

**Fig. 3.**
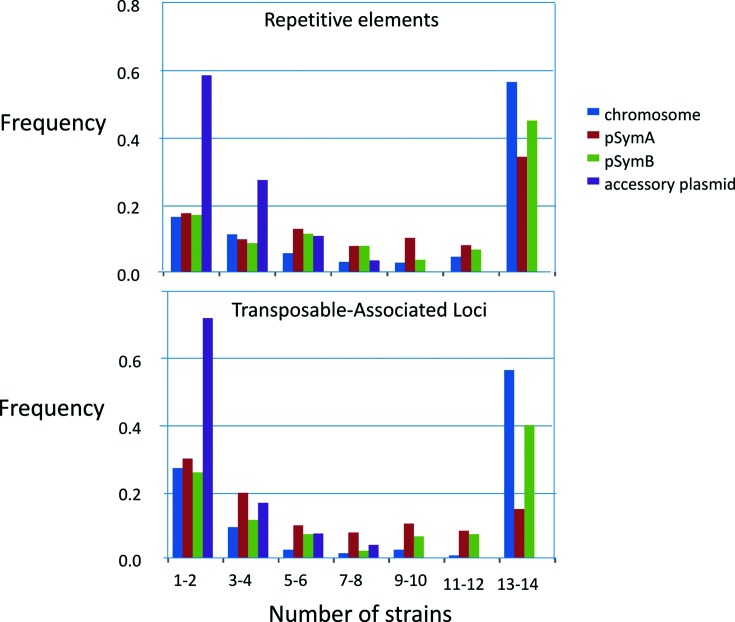
The frequency of occurrence of repetitive and transposable elements found in different numbers of strains. The majority of REs and TALs that are present on accessory plasmids are found on only a single strain, whereas a majority or plurality of REs and TALs found on the chromosome or pSymB are found in all strains. Because of large translocations between plasmids, two strains (USDA1021 and M162) were excluded from analyses leaving a total of 14 primary replicons and 11 accessory plasmids.

Despite being much smaller than the other replicons, the accessory plasmids contained almost half of all the TAL families (Table 1). The TALs on accessory plasmids were also much more likely to be found in only a single strain; more than half of the accessory plasmid TALs were found in only a single strain whereas <25 % of the chromosomal TALs were found in a single strain. In contrast, >50 % of the chromosomal TALs and >40 % of pSymB TALs were found in nearly all of the strains (Fig. 3).

**Fig. 4.**
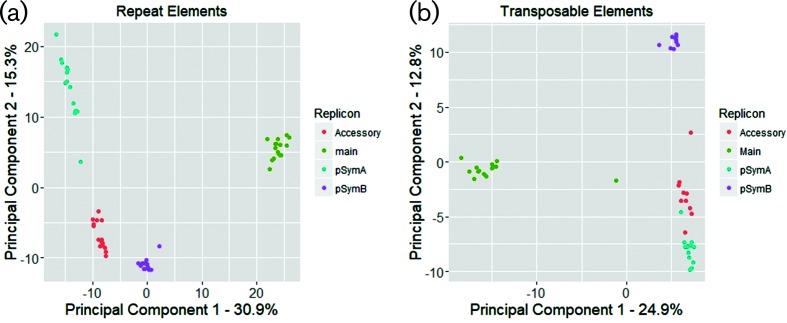
PCA plot of the number of RE (a) and TAL (b) family members on each replicon for each strain. Replicons, rather than strains, primarily cluster together.

A total of 327, 83 and 49 of the TAL families were found on only one, two or three of the main replicons, respectively. If the accessory plasmids were also counted as a single replicon class, 232, 156 and 39 TAL families were found on one, two or three main replicons, respectively. Moreover, 47 TALs were found on all three main replicons and one or more of the accessory plasmids. The presence or absence of specific TALs was more related to a replicon than to a specific strain (Fig. 4).

We also identified repeat sequence elements (REs) by first searching for regions of high similarity within each genome [27], and then clustering these sequences, based on sequence identity, across all genomes to form hidden Markov models (HMMs). This process identified 48 133 repeated elements, ranging in size from 66 to 12 593 bp, that clustered into 688 repeat families by similarity Markov clustering (MCL) [52].

Each strain contained from 2702 to 3132 REs, accounting for 1.46–2.04 Mb of DNA, and comprising ~22–28 % of the total sequence content of each strain. The REs were more evenly distributed amongst the three main replicons than were the TALs: the chromosome contained 417–446 repeats/Mb, while pSymA contained 380–561 repeats/Mb, and pSymB 390–408 repeats/Mb (Table 1). In contrast to TALs (Fig. 3), approximately 50 % of the repeat families were found in all strains, and less than 5 % were found in only a single strain (Fig. 3). Although the majority of REs were not replicon-specific, the distribution of REs among all replicons and strains was more related to a replicon than to a strain (Fig. 4). Like TALs, the REs could be clustered into families with 78, 22 and nine found only on pSymA, pSymB or the accessory plasmids, respectively. The accessory plasmids and pSymA shared 68 RE families that were not found on the chromosome or on pSymB, whereas there were 14 RE families shared by the accessory plasmids and the chromosome but not with pSymA, and five RE families were shared by the accessory plasmids and pSymB but not with pSymA.

### Accessory plasmids as part of the pSymA pan-replicon

The 14 accessory plasmids we identified in our sample contained 3215 predicted genes. These accessory plasmids had a similar ratio of predicted genes, including TALs (1.01 genes/kb), to the other replicons, the chromosome (1.03 genes/kb), pSymB (1.01 genes/kb) and pSymA (1.17 genes/kb). As expected, there were no genes in common among all the accessory plasmids, and several of these plasmids shared little sequence identity with any of the other accessory plasmids. Adding the accessory genome to the pSymA core and pan genome increased the number of core genes from 536 to 553 (excluding M162 and USDA1021, Fig. S4). The RepA protein was not identical across all accessory plasmids based on amino acid comparison (Fig. S5), indicating a potentially diverse source of genetic material [53]. RepA proteins that were highly similar to those found on our accessory plasmids were also found in other rhizobial species, including *E. medicae*, *Ensifer fredii* and *Agrobacterium tumefaciens*.

The gene content of the accessory plasmids was compared to that of the pan-replicon of each of the three main replicons for all 16 strains, based on gene annotation from MaGe (Table 2). The results of this analysis indicated that more genes found on the accessory genome were exclusively found on pSymA, rather than pSymB or the chromosome. This suggests that the accessory plasmids and pSymA have a greater ability to exchange genetic elements than the other replicons. TALs accounted for about 12 % of the total gene content of the accessory plasmids. Less than 2 % of TALs were exclusive to the chromosome or pSymB, whereas 32 % of the accessory plasmid genes were also found exclusively on pSymA.

**Table 2.** Accessory plasmid genes shared with main *Ensifer* replicons

Strain and accessory plasmid names	Total genes	Chromosome-exclusive	pSymA-exclusive	pSymB-exclusive	Multiple replicons
AK83 – plasmid NC_015592.1	107	4	16	1	2
AK83 – plasmid NC_015597.1	391	10	128	4	62
GR4 – plasmid NC_019846.1	213	2	90	1	17
GR4 – plasmid NC_019847.1	374	0	132	2	82
HM006 – accessory plasmid A	248	5	63	2	28
Kh35c – accessory plasmid A	215	3	88	1	24
M162 – accessory plasmid A	497	1	359	1	37
M270 – accessory plasmid A	431	9	83	3	88
M270 – accessory plasmid B	304	11	17	1	46
M270 – accessory plasmid C	181	4	46	2	22
Rm41 – accessory plasmid A	277	3	10	2	12
T073 – accessory plasmid A	197	6	50	0	10
USDA1157 – accessory plasmid A	353	1	216	2	38
USDA1021 – accessory plasmid A	379	13	152	1	87

Further evidence of a shared pSymA/accessory plasmid genome was found by performing a blast analysis against the NCBI NT database using each of the accessory plasmids as a query sequence. Many of the accessory plasmids had blast alignments to plasmids found in other rhizobial species, and many contained genes similar to those found on pSymA (Table S4). For example, the accessory plasmid from strain T073 contained a 13 kb gene region matching *E. fredii*, an accessory plasmid from *Ensifer sojae* and plasmids A and C in *Ensifer americanum* at ~95 % identity.

Perhaps the most interesting match found was between accessory plasmid B in strain M270, which contained 64 genes (>20 % of the total) that were also found on the Ti-plasmid in *A. tumefaciens* strain C58 (Fig. S6, Table S5). Genes involved in agrocinopine synthases, transport and catabolism were present, but those required for T-DNA transfer [54] were missing. Agrocinopines are a sugar-phosphodiester subclass of opines (amino acid derivatives) that are typically found in tumours induced by *A. tumefaciens* [55]. While agrocinopines were originally thought to only be synthesized in crown gall tumours, a wide variety of bacteria are capable of utilizing them [56], and *E. meliloti* strain M270 probably gained the ability to synthesize and catabolize these opines via horizontal gene transfer. Additionally, the genes involved in conjugation were also present. These genes were also found on other accessory plasmids, which may indicate a transmission advantage for accessory plasmids with a conjugation gene cluster.

### Replicons have distinct evolutionary lineages

A Mantel test was used to determine if the nucleotide content of the replicons (chromosome, pSymA and pSymB) in a specific strain diverged together or separately. The Mantel test revealed no statistically significant correlations in pairwise divergence among strains for any pairs of genes on replicons (all *P*>0.3, Table S6, Fig. S7). This shows that two strains might have a similar chromosome but distinct pSymA or pSymB replicons. The results in Fig. S8 show that the phylogenetic trees of single-copy core genes for each replicon were distinct. Additionally, the Microscope Gene Phyloprofile tool was used to examine the proportion of genes found in each strain as compared to the reference strain Rm2011 (Table S7). The lack of correlation in the rate of divergences can be seen by comparing the order of strains. For example, strain T073 had the fourth most similar chromosome, but was only the 14th closest to pSymA when compared to Rm 2011. In contrast, strain Rm41 had the 12th most similar chromosome and was the fourth closest to pSymA.

## Discussion

Characterizing genomic diversity is an important step in identifying genes responsible for naturally occurring variation, as well as gaining insight into past adaptation and evolutionary processes. The *E. meliloti* genome consists of three large replicons found in every strain, as well as smaller accessory plasmids that were present in only some strains. Previous analyses found that the replicons differ in their evolutionary histories, due to the strength of purifying selection to which they are subjected, the extent of horizontal gene transfer and the proportion of core versus accessory genes [5, 10]. Here, we show that the three primary replicons also do not often exchange genes with each other, leading to replicons with distinctive G+C, gene, RE and TAL contents.

Perhaps most strikingly, we saw evidence for only 40 non-transposable-element core genes having moved between replicons – fewer than 1 % of the 4600 core genes, with the exception of a single large translocation event involving the movement of a ~300 kb region from the chromosome to pSymB. The nearly complete lack of core gene movement between replicons is puzzling given that there is experimental evidence for frequent genome rearrangements in *Ensifer* under laboratory conditions [57], and some closely related rhizobia have shown genome instability and plasticity. Additionally, essential genes from an *Ensifer* ancestor are thought to have moved from the chromosome to pSymB [58].

TALs and REs, both of which are able to contribute to gene movement through the translocation of genes between cells and mediate horizontal gene transfer, are abundant on each replicon and often found on multiple replicons [15, 59–62]. We identified a relatively large numbers of REs and TALs compared to many bacterial genomes. The main chromosome had ~18 % repeated sequence in our population. In another study prokaryotic genomes with 20 %+ repeated sequence had the highest repeat coverage from a sample of 720 genomes [63]. Our study was conducted using with different metrics than those used by Treangen *et al*. [63], and probably identified more repeated sequences to identify more diverged repeated elements.

The presence of TALs and REs on multiple replicons indicates that movement between replicons is possible, as do the two large translocation events we detected – a ~300 kb region that moved from pSymA to an accessory plasmid, and a 325 kb region that moved from the chromosome to pSymB. Although the G+C content consistently differed among replicons, a phenomenon also found for the multiple chromosomes in *Burkholderia cenocepacia* [64], the magnitude of the difference (<3 %) is not expected to act as an appreciable barrier to gene exchange. The TALs, unlike the REs, did cluster more closely between pSymA and the accessory plasmid (Fig. 1, Table 1), suggesting that TALs may be involved in inter-replicon gene transfer between pSymA and accessory plasmids.

Understanding inter-replicon gene movement is also important to understand the evolution of *Ensifer* strains as foreign DNA inside the cell will either be eliminated, persist autonomously as a plasmid, or become co-integrated into an existing plasmid through inter-replicon gene transfer [65]. Although TALs can clearly have important roles in gene movement, only seven of 40 possible inter-replicon translocation events we detected had TALs in the regions flanking the translocated regions. In contrast, a high frequency of rearrangements has been found in *Rhizobium etli* [66, 67], and all of the genes showing evidence of inter-replicon movement were found in multiple copies, in at least some strains. This suggests that gene duplication may be involved in inter-replicon gene transfer between replicons in *Ensifer*. Another possibility is that gaining a functionally redundant gene on a different replicon allows for the loss of the original gene without loss of critical function.

Despite finding that <1 % of core genes are found on different replicons in different strains, we detected evidence for extensive gene movement within replicons, and within the chromosome and two megaplasmids. We also found that movement of genes between pSymA and the accessory plasmids was great, with >40 % of accessory plasmid genes also found on pSymA. Given that small plasmids can play central roles in inter-strain gene transfer through conjugation [62], the high rate of gene sharing between pSymA and accessory plasmids suggests that these accessory plasmids might be an important mechanism by which genes are moved between strains, and potentially other bacterial species. This is particularly important from a symbiotic and host-range perspective, given that many of the genes that are essential for establishing a functional symbiosis are found on pSymA [68].

Accessory plasmids in *E. meliloti* have been shown to cause host incompatibility or increase nodulation competitiveness [9]. Indeed, the type IV secretion system, which has been shown to have a variety of effects on nodulation [69, 70], is found on an accessory plasmid or pSymA [69]. Because accessory plasmids can exchange gene content with pSymA, this may allow for the rapid gain or loss of symbiosis-related genes. This phenomenon may lead to some of the symbiotic instability noted for these and other fast-growing rhizobia, where symbiosis genes are plasmid-borne.

While genes on the accessory plasmid were also found as part of the pSymA pan-genome, this was not the case for all genes and some were found in bacterial species other than rhizobia, presumably the result of horizontal gene transfer. Most striking was that one of the *E. meliloti* accessory plasmids had 64 *A. tumefaciens*-like genes. This indicates that rhizobia can probably obtain genes from *Agrobacterium* and other soil microbiota in their free-living, saprophytic, soil phase of existence. *Agrobacterium* and *Ensifer* are closely related and represent different genera within the family *Rhizobiaceae* [71]*. Agrobacterium* Ti-plasmids can be maintained and expressed by *E. meliloti*, although this rhizobial transconjugant is still unable to form tumours on plants [72] although *Rhizobium trifolii* can induce tumours with the addition of a Ti-plasmid [73]. Although the *Ensifer* M270 accessory plasmid B did not contain the tumour-inducing genes, it did contain opine metabolism genes that are found in *A. tumefaciens* Ti-plasmids [74]. This may give this bacterium a selective advantage for growth in some soils and in association with plant roots.

### Conclusion

The complete sequences and analyses of 16 *E. meliloti* genomes offer important insight into the evolution of symbiosis-related loci in this bacterium. Our analyses, done using *de novo* assembled long read sequence data, revealed that the three main replicons have different characteristics with respect to gene content, REs, TALs and G+C content. Ten of the strains harboured accessory plasmids, often with distinct replication proteins, and their gene content was more similar to that of pSymA than to the other replicons. Further studies should investigate this phenomenon, which may give insight into how accessory plasmids form and interact in populations of rhizobia in soils. Intra-replicon gene transfer is associated with REs, but not TALs. The gene transfer events that occurred between the accessory plasmids and pSymA demonstrate one mechanism by which the *Ensifer* symbiosis is constantly evolving.

## Data bibliography

1. DNA sequences have been deposited to NCBI under bioproject number PRJNA388336 (2017). Sequences used in this study were also obtained from:
2. Galibert F, Finan TM, Long SR, Puhler A, Abola P *et al*. The composite genome of the legume symbiont *Sinorhizobium meliloti*. DOI: 10.1126/science.1060966 (2001).
3. Galardini M, Pini F, Bazzicalupo M, Biondi EG, Mengoni A. NCBI Bioproject PRJNA41993, PRJNA42477 (2013).
4. Sallet E, Roux B, Sauviac L, Jardinaud MF, Carrère S *et al.* NCBI Bioproject PRJNA187276 (2013).
5. Martínez-Abarca F, Martínez-Rodríguez L, López-Contreras JA, Jiménez-Zurdo JI, Toro N. NCBI Bioproject PRJNA175860 (2012).
6. Schneiker-Bekel S, Wibberg D, Bekel T, Blom J, Linke B *et al.* NCBI Bioproject PRJNA41117 (2011).

## Supplementary Data

2811 Supplementary File 3

## Supplementary Data

83 Supplementary File 2
