# Supplementary material for: The complete replicons of 16 Ensifer meliloti strains offer insights into intra- and inter-replicon gene transfer, transposon-associated loci, and repeat elements
Source: Microb Genom. 2018 Apr 19;4(5):e000174. doi: 10.1099/mgen.0.000174 (PMC5994717; doi:10.1099/mgen.0.000174)
Supplement: Supplementary File 3 [file mgen-4-174-s001.pdf]

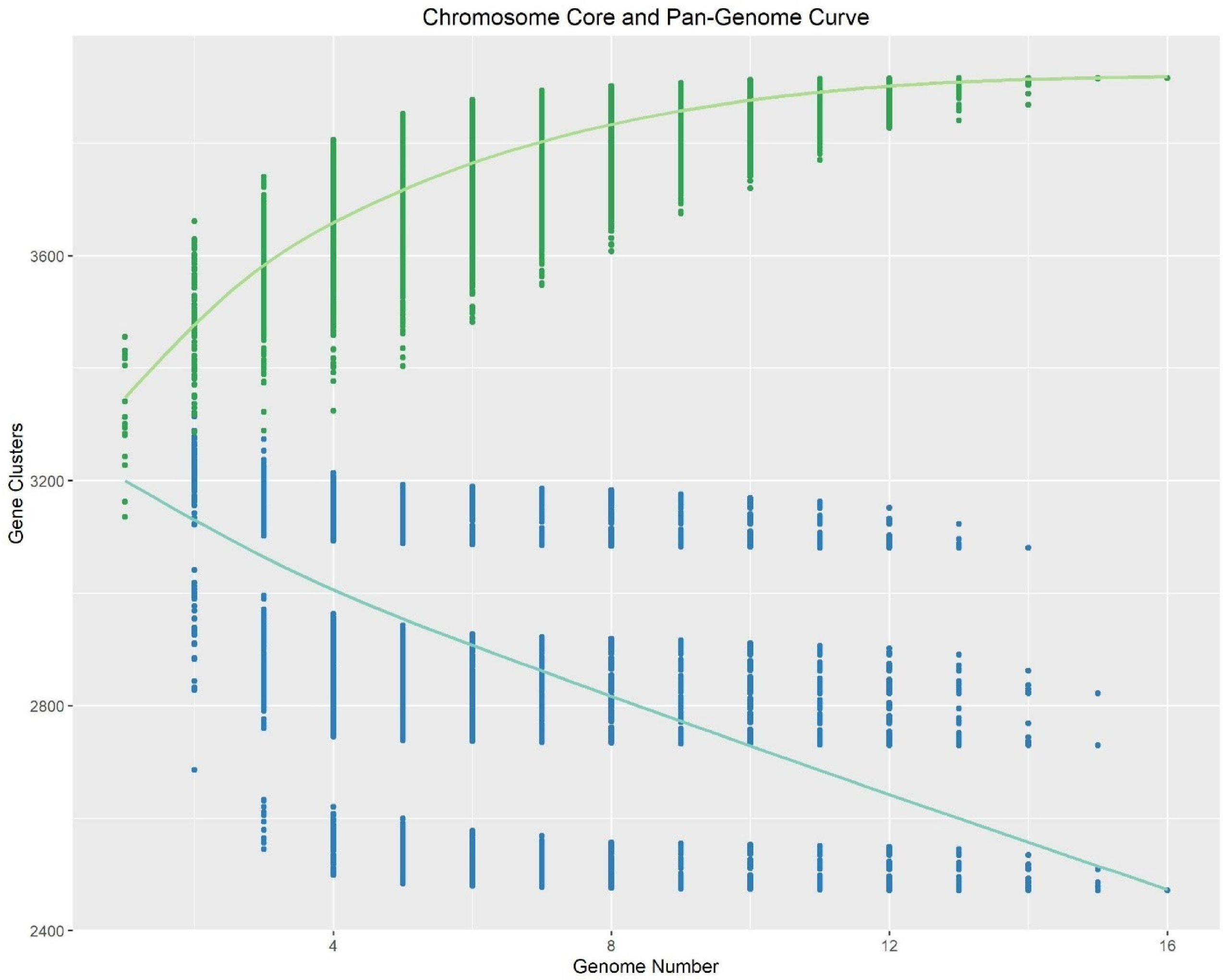


Supplemental Figure 1: Core and Pan-Genome Curves for the main chromosome of our 16 *E. meliloti* strains. Each point represents a combination of different genomes. Blue represents core genome curve, with a combination of our 16 genomes specified by genome number. Genomes are represented by distinct gene clusters, rather than individual genes.

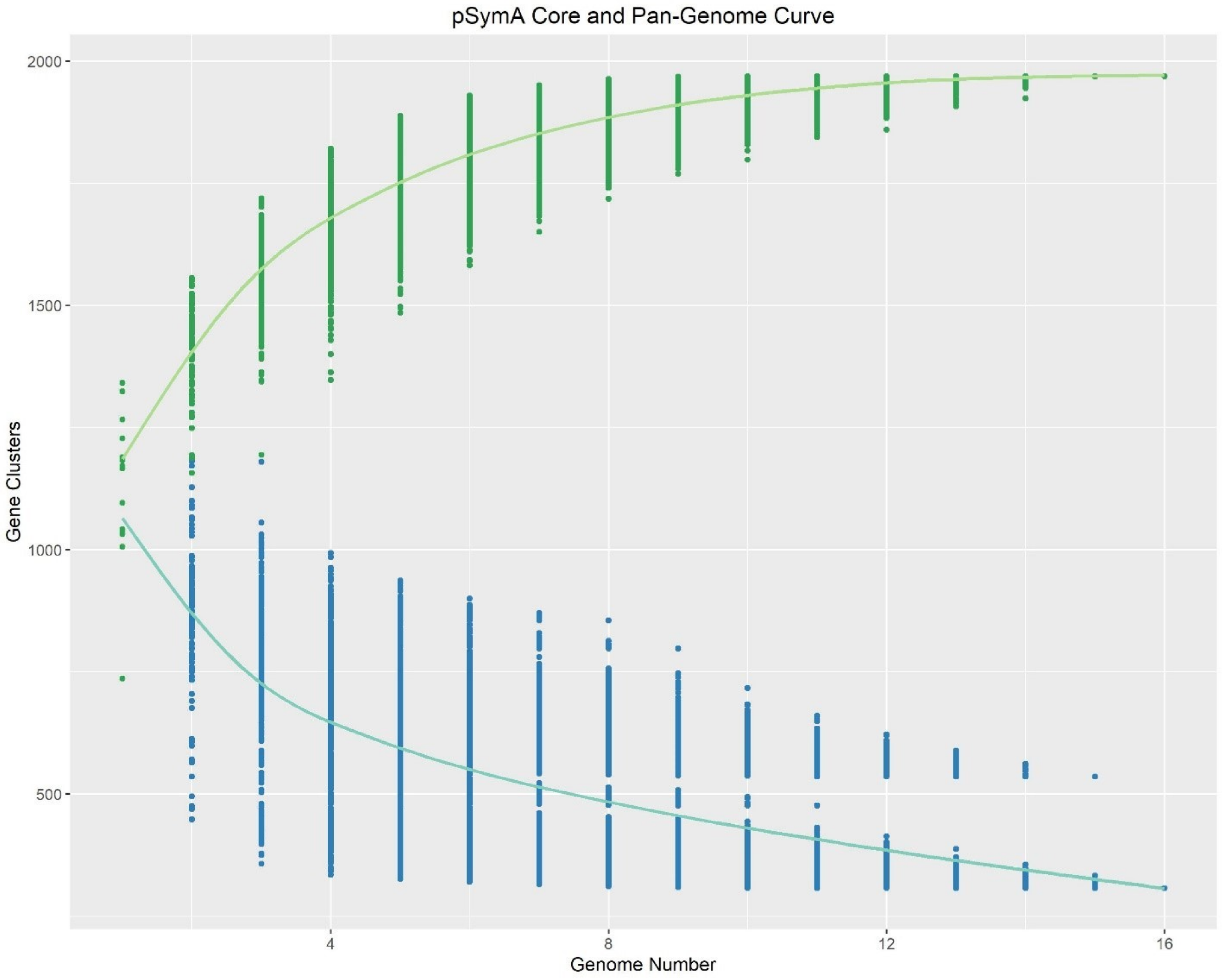


Supplemental Figure 2: Core and Pan-Genome Curves for the pSymA replicon of our 16 *E. meliloti* strains.

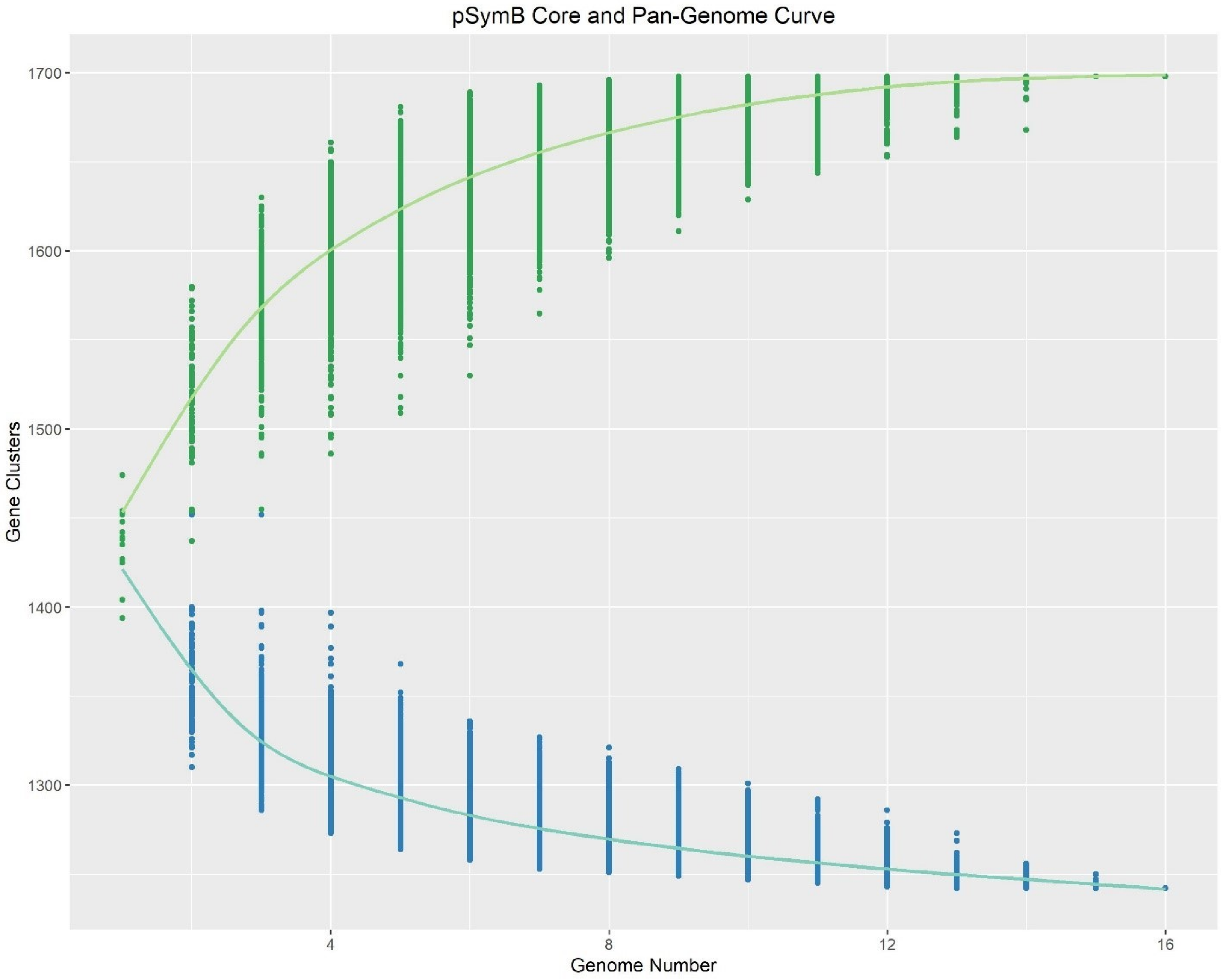


Supplemental Figure 3: Core and Pan-Genome Curves for the pSymB replicon of our 16 *E. meliloti* strains.

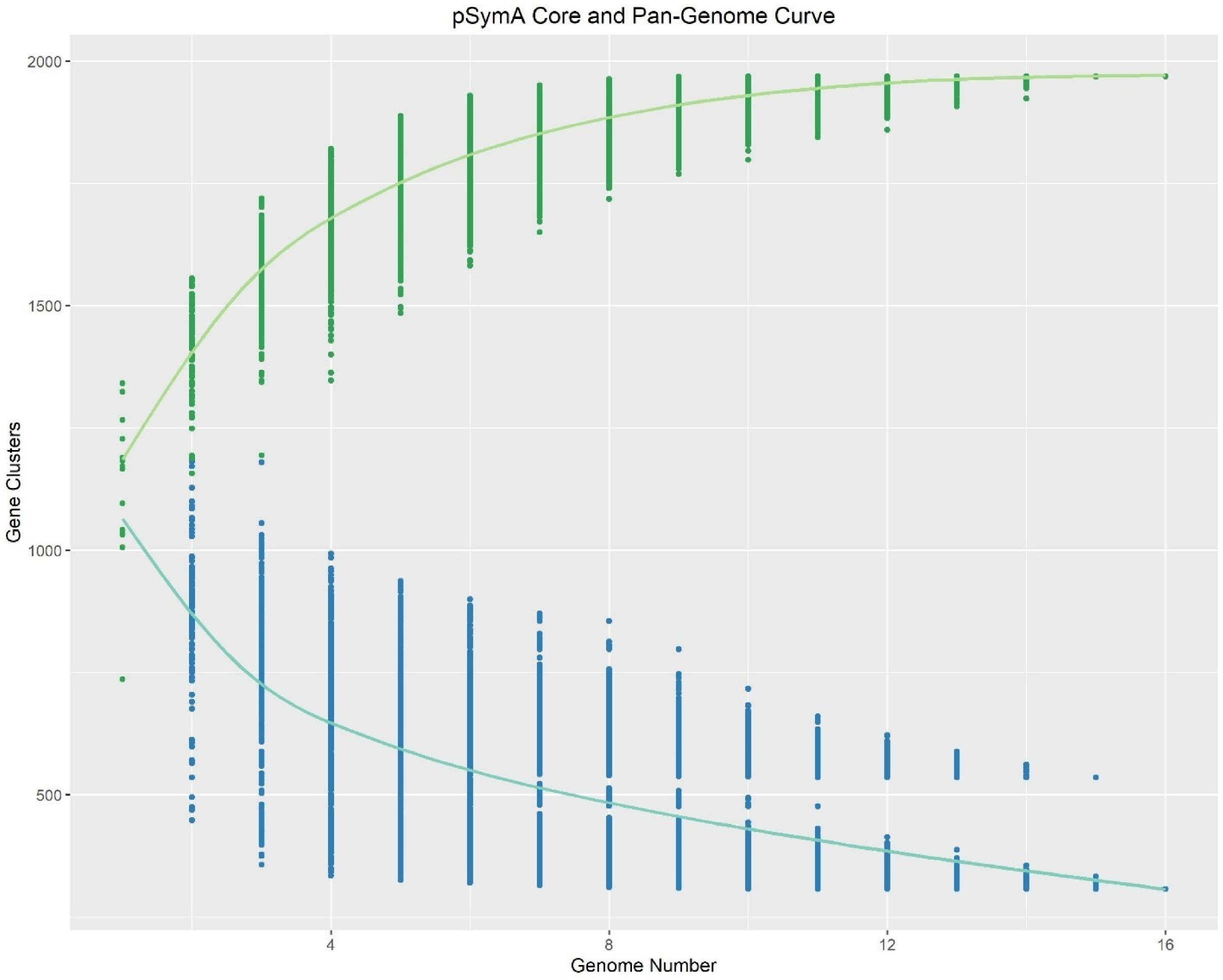


Supplemental Figure 4: Core and Pan-Genome Curves for pSymA and accessory plasmids of our 16 *E. meliloti* strains. The core genome increases with the addition of the accessory plasmids. The pan-genome curve is similar which is an effect of pSymA and the accessory plasmids having genes in common as well as the small size of the accessory plasmids, contributing far less genes to the pan-genome than pSymA replicons.

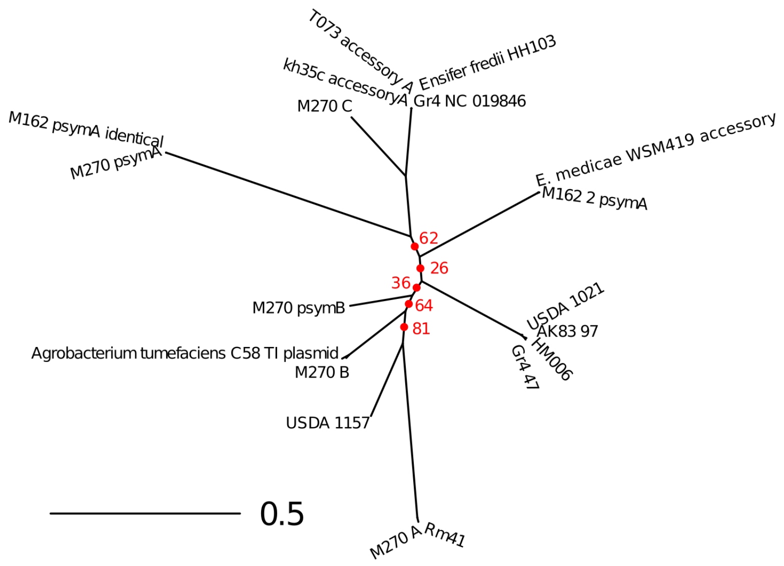


Supplemental Figure 5: Amino acid neighbor joining unrooted phylogenetic tree of RepA proteins found on the accessory plasmids. Red numbers mark the bootstrap support for any split with <95% support.

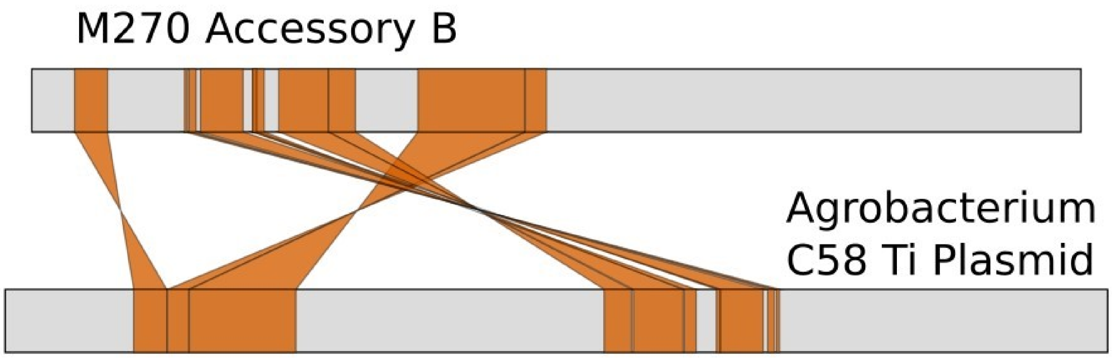


Supplemental Figure 6: Displayed is a synteny plot between Agrobacterium C58 Ti Plasmid and the accessory plasmid B in strain M270. Sequence matches displayed are at least 500bp in length and ID >= 80%.

Supplemental Figure 7: Scatterplots showing pairwise comparisons between individual replicons based on genetic distance

A.

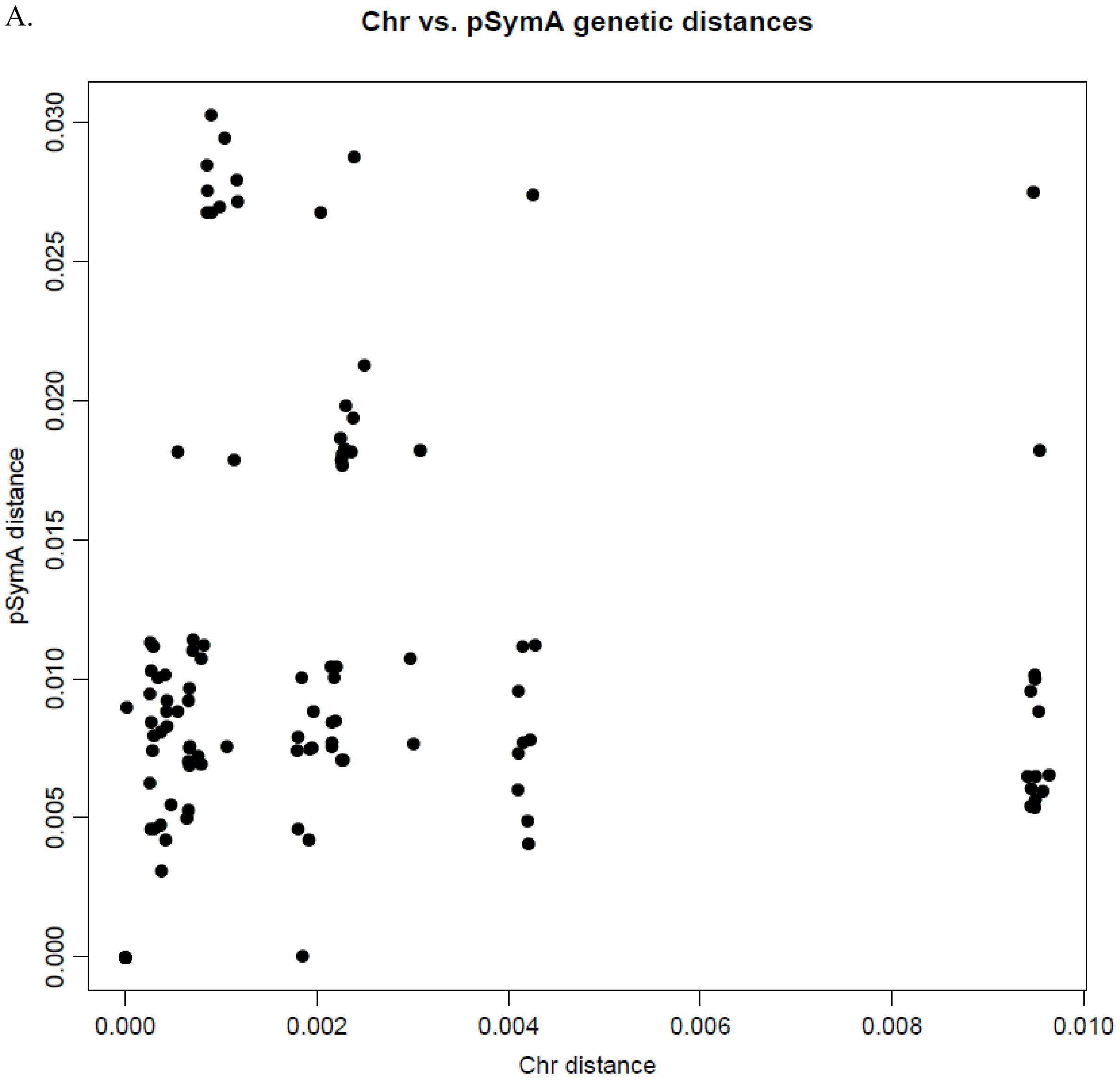

B.

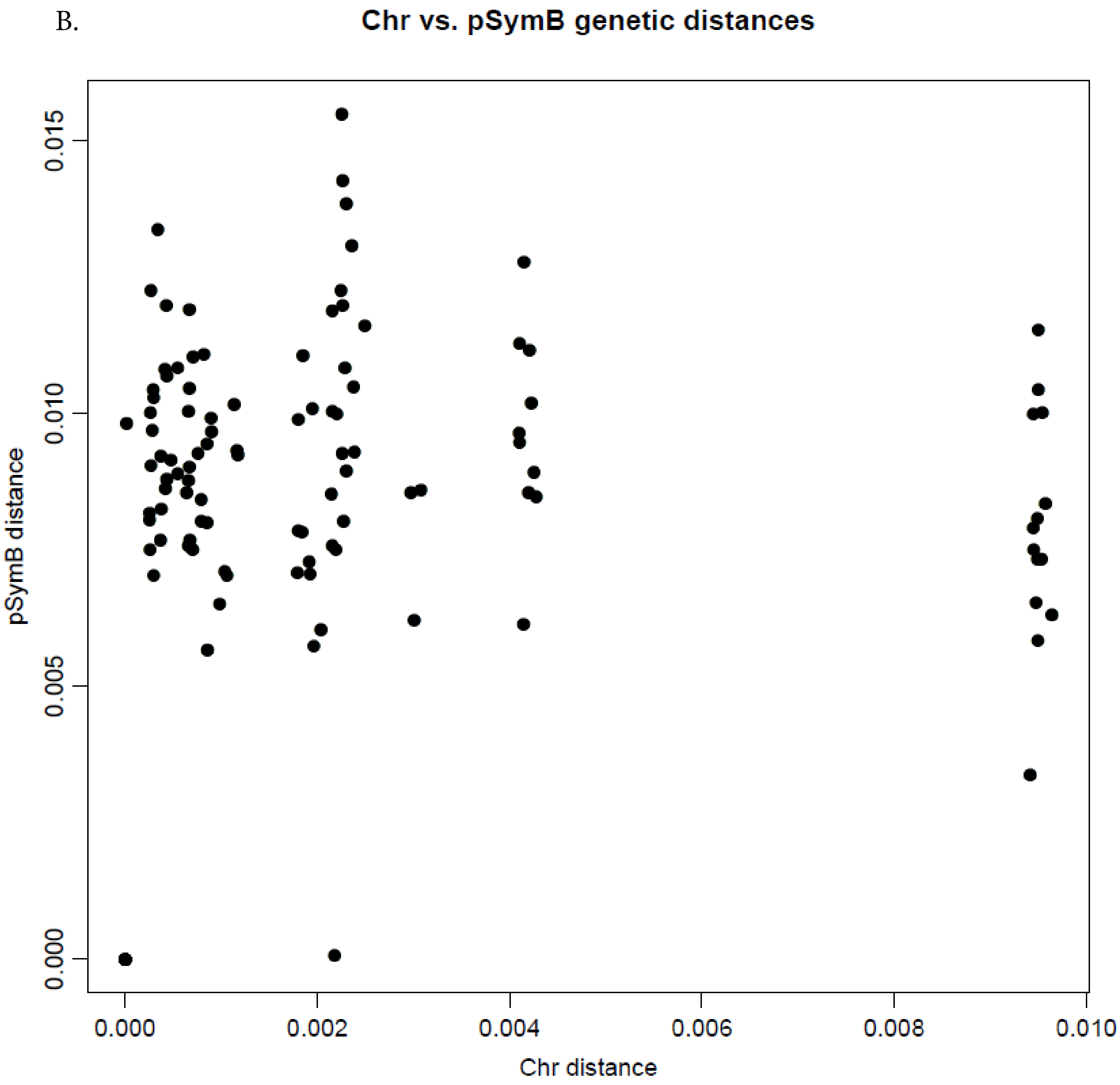
Chr vs. pSymB genetic distances
pSymB distance
0.000
0.005
0.010
0.015
Chr distance
0.000
0.002
0.004
0.006
0.008
0.010

C.

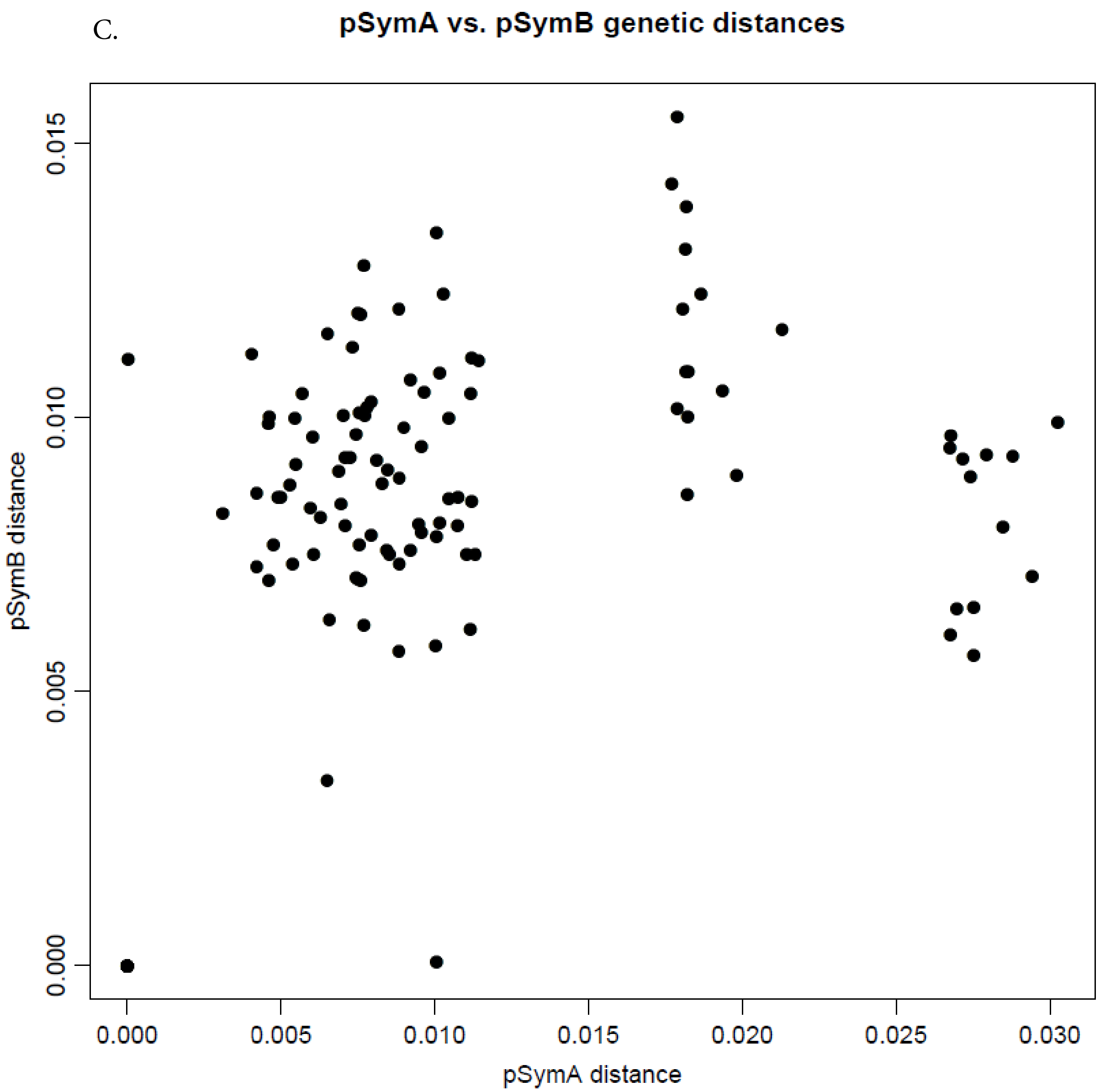
pSymA vs. pSymB genetic distances
pSymB distance
0.000
0.005
0.010
0.015
0.000
0.005
0.010
0.015
0.020
0.025
0.030
pSymA distance

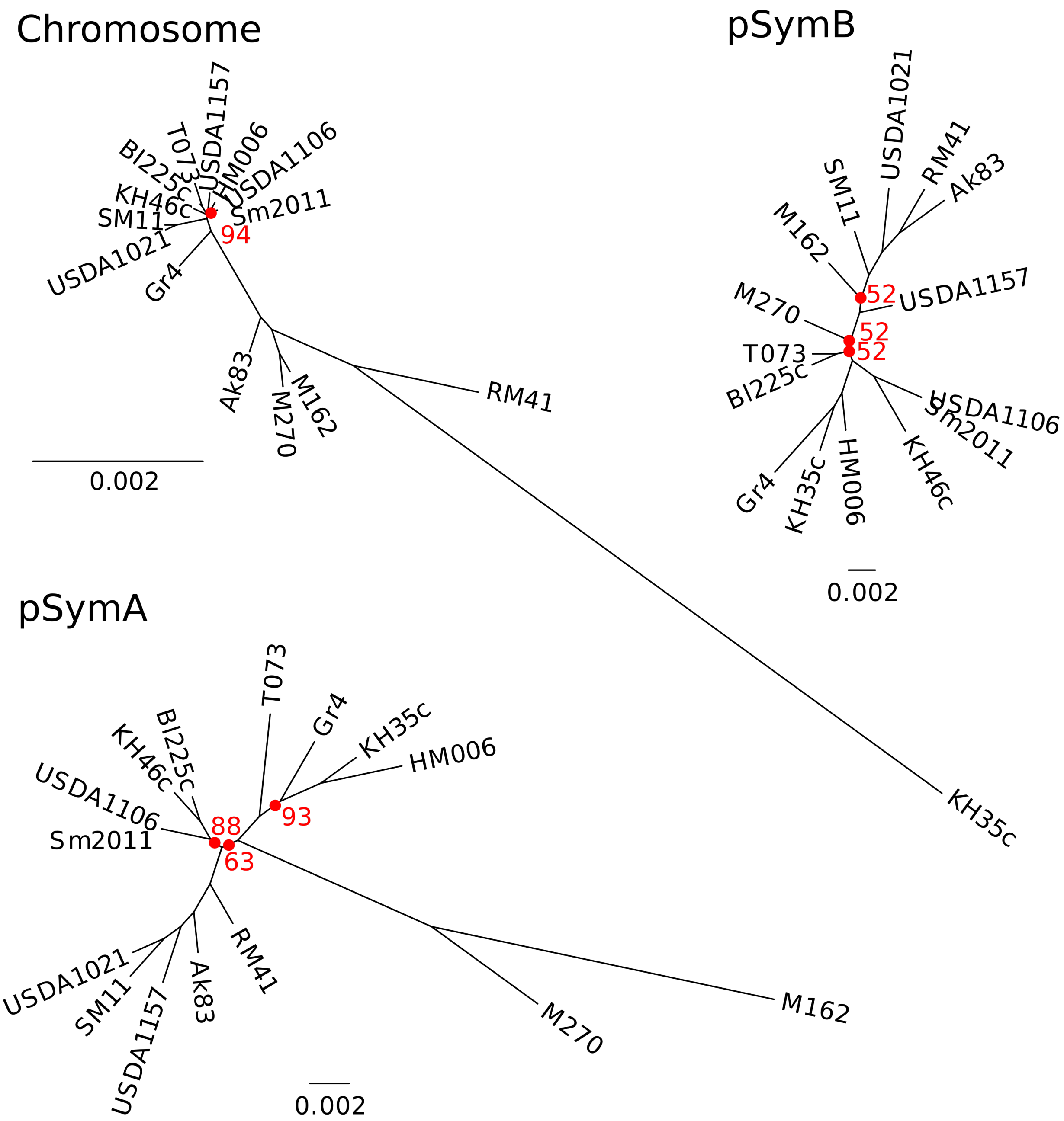


Supplemental Figure 8: Maximum-likelihood phylogenetic trees for each replicon based on the single-copy core genes. Red numbers mark the bootstrap support for any spit with <95% support.
